# Trabecular meshwork's collagen network formation is inhibited by non‐pigmented ciliary epithelium‐derived extracellular vesicles

**DOI:** 10.1111/jcmm.16408

**Published:** 2021-03-01

**Authors:** Saray Tabak, Sofia Schreiber‐Avissar, Elie Beit‐Yannai

**Affiliations:** ^1^ Clinical Biochemistry and Pharmacology Department Ben‐Gurion University of the Negev Beer‐Sheva Israel

**Keywords:** collagen, extracellular vesicles, non‐pigmented ciliary epithelium, open‐angle glaucoma, trabecular meshwork

## Abstract

The present research aims to determine whether the application of non‐pigmented ciliary epithelium cells derived extracellular vesicles to human trabecular meshwork cells affects the formation and secretion of collagen type I to the extracellular matrix formation. Following the extraction of non‐pigmented ciliary epithelium derived extracellular vesicles by a precipitation method, their size and concentration were determined using tunable resistive pulse sensing technology. Extracellular vesicles were incubated with trabecular meshwork cells for 3 days. Morphological changes of collagen type I in the extracellular matrix of trabecular meshwork cells were visualized using confocal microscopy and scanning electron microscopy. A Sirius Red assay was used to determine the total amount of collagen. Finally, collagen type I expression levels in the extracellular matrix of trabecular meshwork cells were quantified by cell western analysis. We found that non‐pigmented ciliary epithelium extracellular vesicles were very effective at preventing collagen fibres formation by the trabecular meshwork cells, and their secretion to the extracellular matrix was significantly reduced (*P* < .001). Morphological changes in the extracellular matrix of trabecular meshwork cells were observed. Our study indicates that non‐pigmented ciliary epithelium extracellular vesicles can be used to control collagen type I fibrillogenesis in trabecular meshwork cells. These fibrils net‐like structure is responsible for remodelling the extracellular matrix. Moreover, we suggest that targeting collagen type I fibril assembly may be a viable treatment for primary open‐angle glaucoma abnormal matrix deposition of the extracellular matrix.

## 1 INTRODUCTION

Primary open‐angle glaucoma (POAG), the most common form of glaucoma disease, is the leading cause of irreversible blindness worldwide. This age‐ and race‐related optic neuropathy is characterized by a gradual loss of retinal ganglion cell neurons, currently affecting over 60 million individuals.^1^, ^2^, ^3^ Several studies have identified elevated intraocular pressure (IOP) as a primary risk factor for the onset and progression of POAG.^4^, ^5^, ^6^, ^7^ Even though the pathophysiology leading to elevated IOP in affected patients is not fully understood, maintenance of IOP is of critical importance to preserving the overall function and health of the retina.^8^ In healthy eyes, IOP is maintained through balanced production and outflow of aqueous humour (AH).^9^ The non‐pigmented ciliary epithelium (NPCE) is the site of AH production, located in the anterior chamber of the eye.^10^ Under homeostatic conditions, the majority (>50%) of AH exits the adult human eye through a conventional outflow pathway, consisting of trabecular meshwork (TM) cells, juxtacanalicular tissue (JCT), and Schlemm's Canal.^11^, ^12^ TM cells are thought to regulate the AH outflow facility through contraction and relaxation of their actin cytoskeleton, synthesis, and secretion of appropriate extracellular matrix (ECM) components.^11^

TM's ECM supplies a specific micro‐environment with unique biomechanical properties fundamental for its biological function. Collagen types I, III and IV, for example, are found in the TM.^13^, ^14^ The main structural component of connective tissue is collagen. Its degradation process plays a role in cell development, morphogenesis, tissue remodelling and tissue repair.^15^, ^16^ More precisely, under pathological conditions disproportionate accumulation of ECM components, such as collagen type I^17^, ^18^ and reduction in hyaluronic acid production, contribute to a decrease in the AH outflow facility leading to elevated IOP.^19^ As a response, TM cells express^13^ and secrete certain molecules such as matrix metalloproteinases (MMPs), in high levels to maintain open flow channels in the JCT.^20^ Thus, ECM degradation takes place mainly by MMPs enzymes.^15^ MMP 2 and MMP 9 are involved in TM ECM metabolism and have been shown to increase aqueous outflow facility.^13^, ^21^

Whereas the underlying cause of POAG remains unclear, one of the theories suggests that signal transmission involving autocrine, endocrine and exocrine processes between tissues is involved in the production and drainage of the AH.^22^ Regarding the ocular tissue, it was reported that exosomes are the major extracellular vesicle population in AH^23^ and may transfer esRNAs (defined as RNA which is shuttled between cells via exosomes) from the NPCE to the TM. AH production, drainage and IOP homeostasis can be synchronized by this esRNA transfer.^24^

Extracellular vesicles (EVs) in general, and exosomes in particular, are nanoscale secreted membrane vesicles, cup‐shaped, in size of 40‐150 nm.^25^ EVs contain materials that mirror the genetic and proteomic content of the maternal cell^26^ such as non‐coding RNA, single‐strand DNA, lipids, proteins and antigen‐presenting molecules.^27^ Researchers have successfully demonstrated that beyond the classical secretory pathways, exosomes play an important role in cell‐cell communications^28^ in close and distant tissues by the delivery of their biological cargo.^29^ EVs are part of the cell response both in normal physiology and in disease pathogenesis.^30^ Moreover, In vitro studies in our laboratory have demonstrated the important role of EVs as signal mediators in NPCE‐TM communication.^30^, ^31^, ^32^ We previously described a general dose‐response at the gene level on MMPs 2 and 9 activities in TM cells depending on NPCE EVs doses.^30^

In a previous study, we detected changes in the expression of two key canonical Wnt signalling proteins in TM cells: pGSK3β and β‐catenin. The phosphorylation of GSK3β allows the release of dephosphorylated β‐catenin from its inactive complex, to translocate into the cell nucleus where it can induce translation of mRNA involved in cell adhesion such as LEF1, Axin2 and β‐catenin.^30^, ^33^, ^34^ We showed that when a relatively low number of NPCE‐derived EVs were incubated with TM cells, a significant decrease in the expression of pGSK3β and β‐catenin was found, while the exposure of TM cells to 10 times higher concentration of NPCE EVs resulted in the abolishment of the down‐regulation expression induced on TM pGSK3β and β‐catenin.^35^ Elevation of cytosolic β‐catenin contributes to the increase in the expression levels of the adhesion molecule, cadherin in general and TM cytoplasm,^31^, ^36^, ^37^, ^38^, ^39^ which is manifested in the development of POAG.^40^, ^41^

Several papers demonstrated that EVs have a role in ECM formation as specific components of their cargo are responsible for changes in collagen fibre formation, and adhesion assembly^42^ in different cells^43^, ^44^ and particularly in the TM.^45^

These findings intrigued us to delve into the question: Do NPCE EVs on TM cells through the canonical Wnt pathway affect the expression and secretion of proteins in the ECM at a later point in time? To do so, we chose collagen type I as the protein of interest.

Monitoring of the morphological changes of collagen and quantification of the expression levels of protein secreted into the ECM are rarely documented. We found that Sirius red reagent^46^, ^47^ and on‐cell western assay provide sensitive and specific tools for the determination of collagen type I levels in TM cells and in its ECM when directly applied to the cell culture in situ.

Here, we examined whether the exposure of human TM cells in culture to NPCE EVs impairs the formation of collagen type I and its secretion to the ECM.

## 2 MATERIALS AND METHODS

### 2.1 Cell culture

Cells were cultured according to published conditions.^30^, ^31^ Briefly, a human trabecular meshwork (TM) cell line was donated by Alcon Laboratories, and maintained in Dulbecco's modified Eagle's medium (DMEM) containing 10% foetal bovine serum (FBS), 2 mmol/L l‐glutamine, 100 μg/mL streptomycin and 100 units/mL penicillin (all from Biological Industries, Kibbutz Beit Ha‐Emek) in a humidified atmosphere of 95% air and 5% CO_2_ at 37°C. Human non‐pigmented ciliary epithelial (NPCE) cell line^22^ was kindly supplied by Prof. Miguel Coca‐Prados, Yale University, USA. Cell lines authentication tests were performed at the Genomics Center of Biomedical Core Facility, Technion, Israel, using the Promega GenePrint 24 System, and the results are attached (Appendix A ). To be more precise, two different splits (TM‐10 and TM‐16) of a human TM‐5 cell line and a human NPCE (ODM‐2) cell line were approved by this authentication test. All cell lines were used up to 25 passages. 50%‐100% cell confluence was used through the studies. NPCE cells were cultured in DMEM depleted of FBS‐derived EVs by overnight centrifugation, using Beckman Coulter ultracentrifugation, for 14 hours, 4°C and 100 000 *g*. Supernatant in a volume of 200 mL was collected and transferred to a 200 mL medium containing 2 mmol/L l‐glutamine, 0.1 mg/mL streptomycin and 100 units/mL of penicillin. EVs depleted serum^48^ was used along with all experiments.

### 2.2 Research model

The previous examination of time‐dependent differences in the expression levels of Wnt signalling proteins and genes exposed to NPCE EVs^31^, ^32^ led us to investigate the effect of NPCE EVs on the ECM of TM cells in specific conditions and period to get the maximal effect. To examine the effect of NPCE EVs on the secretion of collagen type I, TM cells were exposed to NPCE EVs in depleted medium. Medium was removed every 24 hours, and fresh EVs were added. Two quantitative methods were used to assess collagen content: Sirius Red and on‐cell western (OCW). All experiments were carried for five days. Using confocal and scanning electron microscopy (SEM), pictures of the culture were taken to track the architectural changes of the collagen fibres secreted by TM cells to the ECM. Untreated TM cells were used as a control in all experiments.

### 2.3 EVs extraction

EVs were extracted from NPCE or TM cells using polyethylene glycol (PEG) 8000 precipitation^49^, ^50^, ^51^ with slight modifications; cell culture conditioned medium was collected, centrifuged at 1500 *g* for 15 minutes at 4°C, to pellet dead cells and cell debris. Precipitation solution was prepared as follows: 100% PEG‐8000, 1 mol/L NaCl, mixed with the conditioned medium 1:5 v/v, respectively, filtered through a 0.22 µm PVDF filter and incubated overnight at 4°C. The mixtures were centrifuged at 1500 *g* at 4°C for 30 minutes to pellet the EVs. The pellet containing the EVs was re‐suspended in PBS 0.1 mol/L, pH = 7.2, and pelleted by ultracentrifugation at 100 000 *g* for 70 minutes at 4°C. The final pelleted EVs were suspended in 1 mL PBS 0.1 mol/L, pH = 7.2, and were stored at −80°C until use.

### 2.4 Tunable resistive pulse sensing (TRPS)

NPCE EVs size and concentration were determined by qNano (Izon Science) instrument, using the Tunable Resistive Pulse Sensing (TRPS) technology with a NP150 membrane (85‐300 nm).^52^ To eliminate contaminating debris, EVs samples were passed through 0.22 µm filters before TRPS analysis. The apparatus was operated at a voltage of 0.48‐0.64 V without pressure. The membrane was stretched to 47 mm. Polystyrene beads at a concentration of 1.2 × 10^13^ beads/mL (110 nm; Izon Science) were used to calibrate size and concentration, following the manufacturer's instructions. Samples were diluted 1000‐fold with PBS 0.1 mol/L buffer, pH 7.2, and measured up to 10 min. The movement of the particle through the membrane is identified as a change in the ionic stream causing current changes. The signal power is proportional to the particle size. According to the number of particles and their velocity at a specific time, the qNano determines the EVs sizes and concentration.

### 2.5 Sirius Red assay

Staining of collagen by Sirius Red was used to quantify total collagen content in TM cell culture. TM cells were seeded in a 24‐well plate to reach the confluence of 50% in the experiment day. TM cells were exposed to a single dose of 2 × 10^7^EVs/7500 cells per well for 3 days. The same proportion of NPCE EVs: TM cells was kept for each day as the experiment progressed, since TM cells number multiplied itself every 24 hours.

Medium was removed every 24 hours, and fresh EVs were added. On the fifth day, cells were washed twice with PBS fixed with Bouin's fluid for 1 hour.^46^ The fixation fluid was removed, and the culture plate was washed by immersion in running tap water for 30 seconds. The culture dish was air‐dried before adding 1 mL Sirius Red dye reagent (Direct red 80, Sigma Aldrich). The dye was dissolved in saturated aqueous picric acid (BDH Chemicals, laboratory reagents) at a concentration of 5 mg/5 mL. Samples were dried in a biological hood for 30 minutes until all water residues evaporated. The cells were stained for 1 hour under mild shaking on a microplate shaker. Thereafter, the dye solution was removed, and the stained cell layers washed with 0.01 mol/L HCl to remove all non‐bound dye. Then, the stained material was dissolved in 200 µL of 0.1 mol/L NaOH using a microplate shaker for 30 minutes at room temperature. The dye solution was transferred to a 96‐well plate, and the optical density was measured by a microplate reader (Thermo Max microplate reader; Molecular Devices) at 540 nm against 0.1 mol/L NaOH as a blank. All samples were assayed in triplicate, and the collagen dyed optical density of treated TM cells with NPCE EVs was compared to untreated TM cells.

### 2.6 Confocal microscopy analysis

To evaluate whether the addition of NPCE EVs to TM cells affects the formation and structure of collagen fibres secreted by TM cells, NPCE EVs were added to TM cells every 24 hours for 3 days. Untreated TM cells were used as control. Sirius Red reagent was used to mark the total collagen of untreated or treated TM cells (as was described at the Sirius Red assay section). Samples were washed with PBS three times and placed on slides. Images were analysed by an FV1000‐IX81 confocal microscope (Olympus) equipped with an 60× objective.

### 2.7 Scanning electron microscopy (SEM) analysis

After fixation with Bouin's fluid and before Sirius Red staining (Sirius Red assay paragraph), morphological observation of the ECM of treated or untreated TM cells with NPCE EVs was done by scanning electron microscopy. Samples on coverslips were air‐dried for 30 minutes at room temperature, coated with carbon and operated at 2 kV, using the VERIOS XHR 460L Scanning Electron Microscope.

### 2.8 On‐cell western (OCW) assay

Tracking collagen type I secretion to the ECM of TM cells following NPCE EVs treatment was done by OCW assay^47^ with some modifications. An amount of 3125 TM cells per well was seeded in a 96‐well plate (µCLEAR^®^, BLACK, CELLSTAR^®^, TC, lid with condensation rings, sterile, Greiner). 24 hours post‐seeding, TM cells were treated with NPCE EVs in a concentration of 1.6 × 10^7^ EVs/mL depleted medium. EVs were added for two additional days in gaps of 24 hours, keeping the same NPCE EVs: TM cells ratio of 1.6 × 10^7^ EVs/6250 cells. On the fifth day, when TM cells reached full confluence, cells were then processed for OCW analysis. Untreated TM cells were used as control. TM cells were washed twice with 100 µL of PBS (0.1 mol/L) and then fixed for 20 minutes with PBS plus 4% paraformaldehyde at room temperature. Post‐fixation, cells were labelled with IRDye 680RD NHS ester (LI‐COR, BARGAL Analytical Instruments) to allow normalization for protein content. Wells were blocked with intercept Blocking Buffer 500 mL (LI‐COR, BARGAL Analytical Instruments) before labelling with rabbit anti‐collagen type I antibody (Rockland Immuno‐chemicals) for 2 hours at room temperature. Bound primary rabbit antibody was detected using IRDye 800CW conjugated goat‐anti‐rabbit IgG (LI‐COR, BARGAL Analytical Instruments). The plate was read on an infrared scanner at wavelengths of 700 and 800 nm (LI‐COR Odyssey CLxta). Quantification of the signals at each wavelength was performed using Odyssey software. The 800 nm signal was normalized to the corresponding NHS ester signal read at 700 nm according to Filla et al.^47^ The data represent the mean of data pooled from three independent experiments ± SD Analysis was performed using Prism software (GraphPad).

### 2.9 Statistics

Data are presented as mean ± standard deviation. Statistical evaluation of Student's *t* test was performed with GraphPad Prism version 7.0 software. All tests were considered significant at *P* < .05.

## 3 RESULTS

To examine the role of NPCE EVs in collagen secretion by TM cells and ECM remodelling, we used various quantifying techniques, relying on previous work conducted in our laboratory of NPCE EVs characterization.^31^ DLS, NTA, TRPS and TEM defined that purified NPCE derived EVs were detected as small rounded 50‐140 nm membrane vesicles. Additionally, Western blot analysis indicated that NPCE derived EVs were positive for classic exosome markers, including Tsg101, Alix and CD81.^31^ Isolated nanoparticles were found in sucrose density fractions typical of exosomes (1.118‐1.188 g/mL sucrose).

### 3.1 NPCE EVs size and concentration measured by TRPS

**FIGURE 1:**
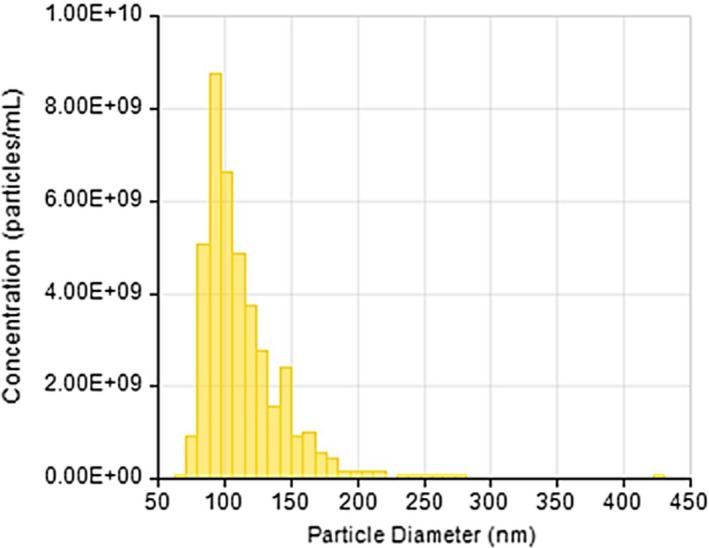
Size and concentration distribution of NPCE‐derived EVs as measured by Tunable Resistive Pulse Sensing Technology (TRPS). A representative bar graph demonstrates a Gaussian distribution of NPCE EVs evaluated from three independent samples

EVs isolated from NPCE cells culture supernatant were measured using TRPS method to determine the size and concentration of these nanoparticles. EVs concentration of 1.3 × 10^7^ particles/mL was used for all experiments. NPCE EVs size was found to be 95.83 ± 10.47 nm in diameter (Figure 1).

### 3.2 Sirius Red staining of collagens

**FIGURE 2:**
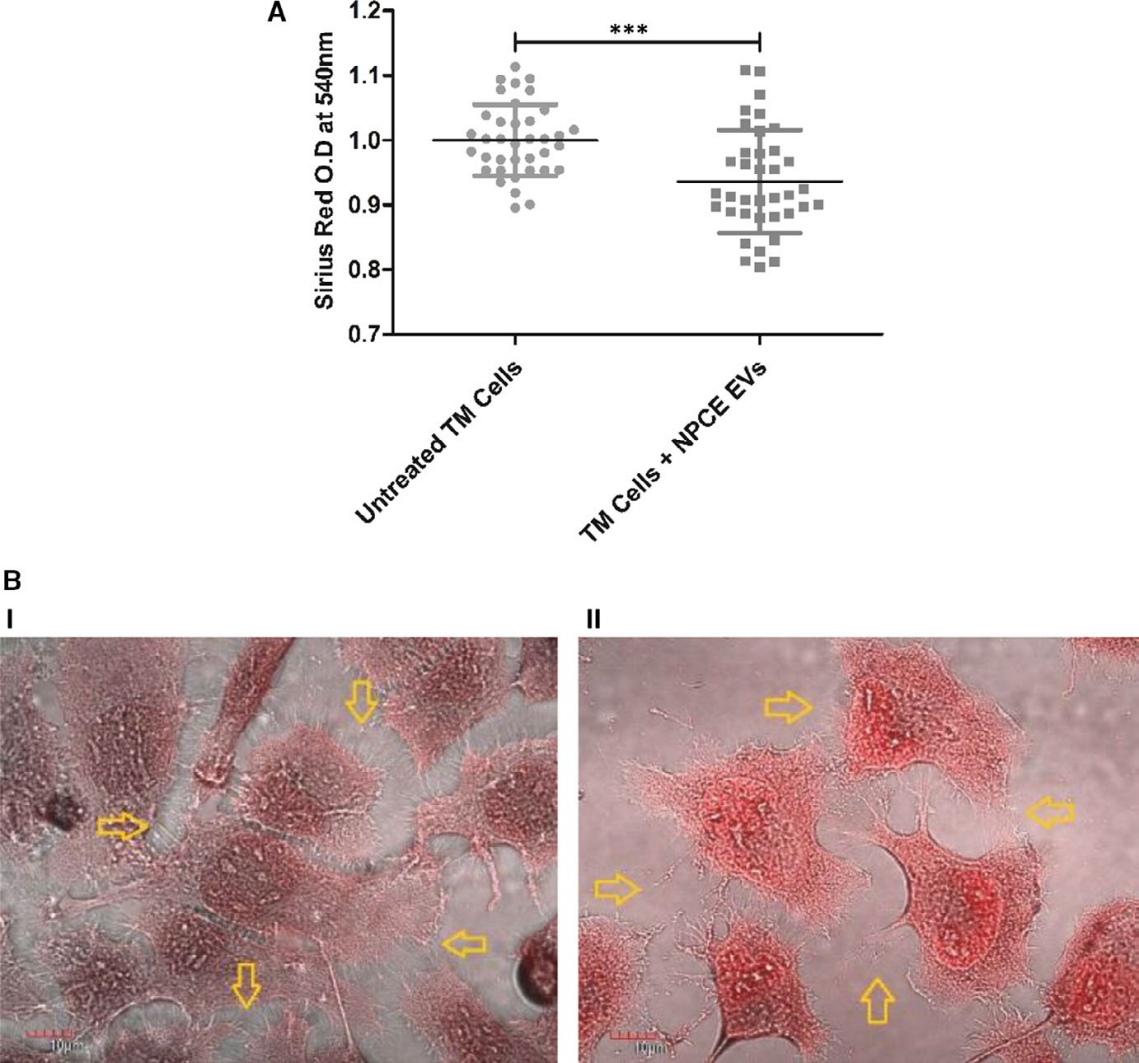
Staining of collagen with Sirius Red reagent. The staining intensities of collagen derived from untreated or treated TM cells with NPCE EVs were determined by ELISA reader at 540 nm. A, The bar graph represents the means ± SD from three independent experiments performed in triplicates. B, Representative confocal images of stained collagen from both untreated (i) or treated (ii) TM cells with NPCE EVs, scale bars are of 10 µm. A minimum of 30 images per treatment was collected. Yellow arrows point to collagen fibrils in the ECM of TM cells. Data represent means ± SD from three independent experiments performed in triplicates. Student's *t* test was used to determine a statistical difference, as indicated by asterisks (***P < .001)

To test the effect of NPCE EVs on collagen formation in the inner and outer milieu of TM cells, Sirius Red reagent was used. The staining intensities of treated or untreated TM cells with NPCE derived EVs, measured at 540 nm, reflect a significant reduction of 6.4% in EVs treated TM cells (***P < .001), suggesting a total decrease in collagen type I content of NPCE EVs treated TM cells (Figure 2A).

### 3.3 Visualization of collagen type I using confocal microscopy

Following Sirius red staining, a qualitative analysis of the NPCE EVs treatment effects on TM cells was conducted by confocal microscopy (Figure 2A). Figure 2B illustrates the effect of NPCE EVs on ECM morphology. TM cells duplication rate (24 hours) was not affected by the addition of NPCE EVs. A lack of collagen fibres and deformation of their original shape can be noticed in the ECM of TM cells treated with NPCE EVs (Figure 2B, II), while the ECM of untreated cells looks denser with abundant collagen fibres (Figure 2B, I).

### 3.4 Tracking the secretion of collagen type I by TM cells using SEM

**FIGURE 3:**
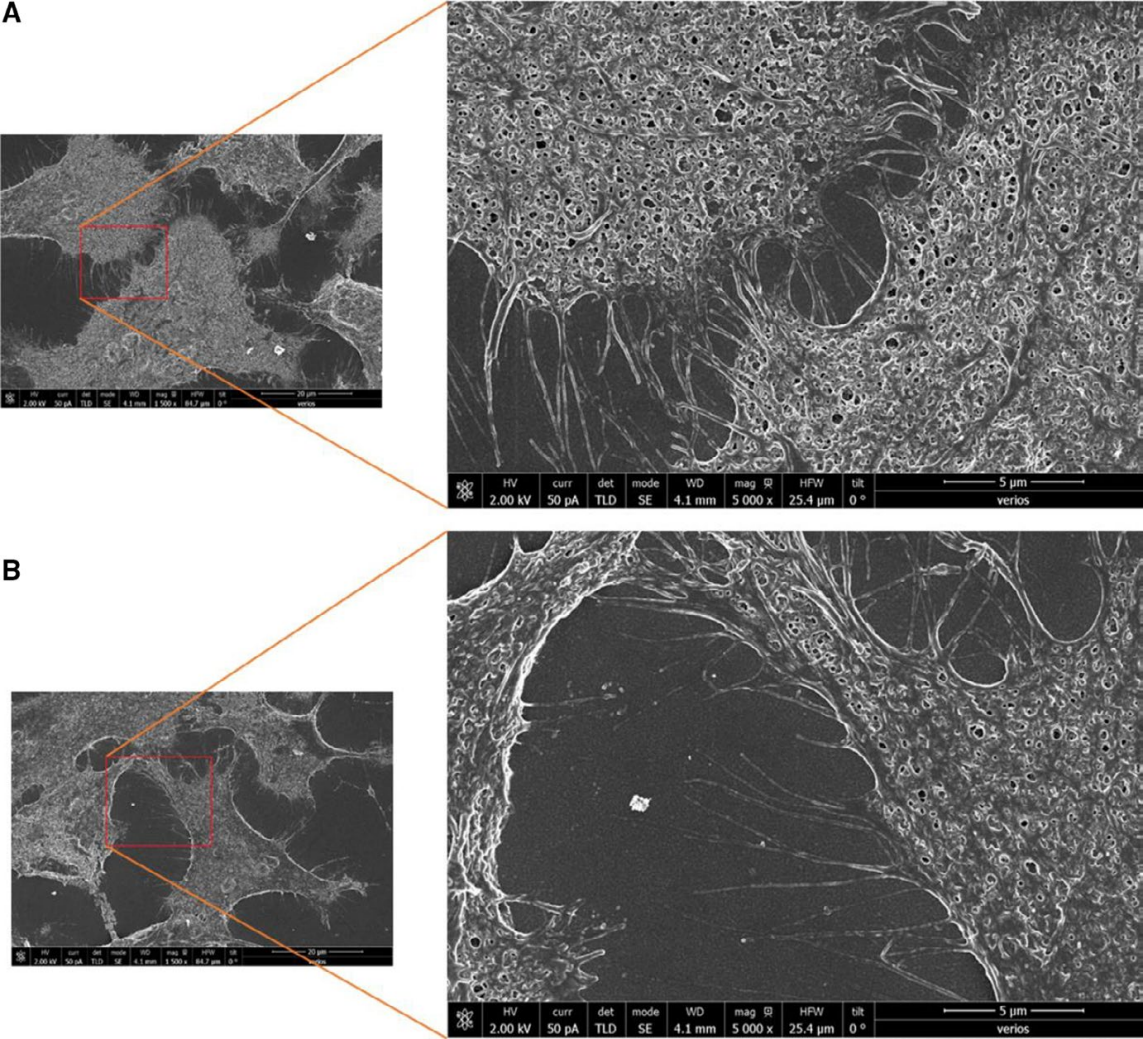
Representative SEM images illustrating the presence or absence of Collagen fibres in the ECM of TM cells. A comparison was made between ECM Collagen fibres of untreated (A) or treated TM cells with NPCE EVs (B). The SEM images magnification are 1500× with the inserts 5000×, scale bars are of 5 µm. A minimum of 30 images per treatment was collected

Additional method for tracking ECM remodelling secreted by TM cells is the scanning electron microscopy (SEM).^53^ In this study, SEM was used for the visualization of Collagen fibres in the ECM of TM cells, (Figure 3). SEM images verified that ECM of TM cells incubated with NPCE EVs for 3 days was less abundant with collagen fibres, and occasional fibres debris were present (Figure 3B) as compared to the untreated TM cells (Figure 3A). Collagen type I forms fibrillar networks and often delineates rounded cavities that presumably contain cells or cell processes in the live specimen.^54^ Collagen fibres of untreated TM cells (Figure 3A) seem to be more elongated and form a network structure in their ECM compared to treated TM cells (Figure 3B).

### 3.5 Quantification of ECM collagen type I secreted by TM cells using on‐cell western (OCW) assay

**FIGURE 4:**
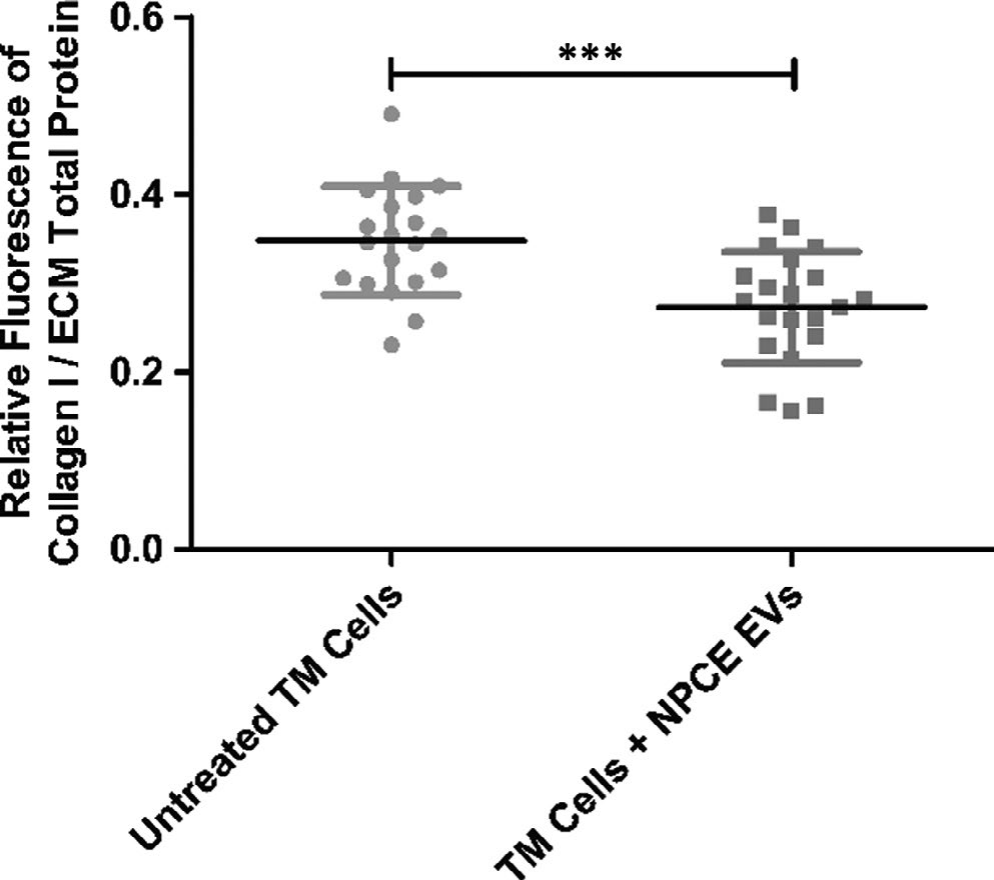
The effect of NPCE EVs on Collagen type I levels in the ECM of TM cells. The plot depicts signal intensities of Collagen type I (800 nm) normalized to ECM total protein (700 nm), obtained in OCW assay from fixed TM cells. Data are means ± SD from three independent experiments performed in triplicates. Student's *t* test was used to determine a statistical difference, as indicated by asterisks (***P < .001)

Unlike the Sirius red reagent used for the determination of total collagen, an OCW assay based on specific antibody recognition of protein in the extracellular milieu was used to measure the levels of type I collagen secreted to the ECM of TM cells treated with NPCE EVs for 3 days. We compared the relative fluorescence intensities at 800 nm, of untreated or treated TM cells with NPCE EVs, using the Licor Odyssey CLx device (Figure 4). Values were normalized to ECM total protein (at 700 nm). A significant decrease in the relative fluorescence of treated TM cells with NPCE EVs was found compared to untreated TM cells (***P < .001).

## 4 DISCUSSION

The overexpression of ECM collagen is considered a major feature of the glaucomatous TM tissue that contributes to the development of outflow resistance.^55^, ^56^ Exposure of human primary TM cells or TM cell line to NPCE EVs has been shown to decrease Wnt protein levels.^30^, ^31^, ^32^ Our previous data support the concept that NPCE EVs biological effects are concentration‐dependent at their target site. We described a different response regarding key canonical Wnt proteins expression that depends on NPCE EVs doses.^30^ Therefore, monitoring β‐catenin levels in the TM cytoplasm may serve as an indicator that can be associated with a beneficial effect of POAG treatment. Further downstream we were able to detect a reduction in pan‐Cadherin protein expression following NPCE EVs treatment of TM cells.^31^ In both studies, we reported on a specific recognition between NPCE EVs and TM cells compared to other EVs types^30^ or cell lines.^31^ For instance, mean fluorescence intensity measurements indicated that there is a specificity of the cellular uptake of NPCE derived EVs by TM cells as compared to retinal pigmented epithelium (RPE) derived EVs.^30^ Moreover, quantitative cellular uptake of labelled NPCE EVs by diverse cell lines after a 12 hours incubation revealed a significant reduction in EVs entry compared to TM cells.^32^ We reported on attenuation of p‐GSK3β and β‐catenin expression levels following uptake inhibitors treatment or EVs membrane proteins removal, thus indicating that Wnt‐TGFβ2 signalling in TM cells is mediated through NPCE EVs surface proteins in an active manner that involves endocytosis‐dependent routes.^57^ Hence, in the current study, only NPCE‐derived EVs were examined.

However, whether NPCE EVs directly influence the downstream, secretion of collagen to the ECM of TM cells is unknown. In this study, we investigated the effect of NPCE EVs on the secretion of collagen type I to the ECM of TM cells.

Collagens I, III, IV, V and VI are present in the cornea and in the TM to variable degrees.^58^, ^59^, ^60^ The formation of standard fibrils that depend on collagens I and III is known to be the classic collagen form. This pattern is visualized throughout the centre of TM beams and JCT region.^17^

Also, type I collagen is a substrate for MMPs 1, 2, 8 and 13. These enzymes affect the outflow facility,^61^ and MMPs 1, 2 and 9 particularly disrupt the collagen network formation.^62^ Characterization of the effects of different NPCE‐derived EVs concentrations, on the activity of MMP 2 and MMP 9 in cultured human TM cells was demonstrated.^30^ In this study, we have found that NPCE EVs have a role in MMP 2 and MMP 9 activity regulation in TM cells, but downstream effects in the ECM of TM cells were not examined.

There is a growing number of studies that employ Western blot and real‐time PCR techniques for collagen levels determination in the cell.^63^, ^64^, ^65^ These methods have limitations for quantifying extracellular collagen. The use of confocal microscopy to visualize collagen by exposure to specific antibodies is also documented in the literature.^66^ However, this method too is not enough quantitative and accurate. Using confocal microscopy raises technical issues, for instance fluorescent *z*‐stacks are limited to a depth of approximately 30‐40 μm because the collagen is too dense for the confocal laser to penetrate any deeper.^67^ Therefore, in the present work, we have used Sirius red and OCW assays.^46^, ^47^ We found these two methods specific for ECM collagen type I reliable quantification, rendering the test useful for large numbers of samples. Since Sirius red dye detects both intracellular and extracellular collagen, and is known to recognize different collagen fibrils within the TM cells or in the ECM, we additionally relayed on OCW technique which is an accurate and specific staining for collagen type I detection using a fluorescent antibody. To overcome the biological limitations of using Sirius red assay, SEM was also used. However, additional supportive experiments are needed to verify the identity of ECM structures shown in Figure 3.

Sirius red (Figure 2A) and OCW (Figure 4) assays demonstrated that NPCE EVs significantly decreased the secretion of type I collagen to the ECM of TM cells. Confocal microscopy (Figure 2B) and SEM (Figure 3) results indicate a morphological alteration in the collagen fibres and their impaired branching in the ECM.

Relationship has been found between daily circadian in the whole animal and the cell cycle, and this relationship has an effect on major cellular processes. Moreover, the cell cycle is manifested mainly in the cell division, but also in the metabolic catabolic processes and post‐translational controls are subject to the influence of the cell cycle. A number of publications have linked the Wnt pathway to the effect of cell circulation and daily circadian^68^, ^69^ related to several diseases. Effects of daily circadian in TM cells focused on Wnt pathway‐mediated IOP changes.^70^ For the NPCE EVs standardization, we collected the NPCE cell medium every 48 hours and combined the entire medium for the extraction process. In general, this was done at about the same time but more important it represents all the EVs released by the NPCE cells along their cell cycle.

In our previous work,^32^ we observed that primary TM cells treated with NPCE EVs (either from either primary or cell line) display a similar trend and power in Wnt signalling attenuation as found in TM cell line. The present findings demonstrate that non‐pigmented ciliary epithelium extracellular vesicles can be used to control collagen type I fibrillogenesis in trabecular meshwork cells an essential step in ECM remodelling.

In summary, the data presented here suggest that collagen secretion to the ECM and its formation by TM cells is at least partially regulated by NPCE EVs. These are important observations that should influence future glaucoma research, focusing on facilitating AH outflow and lowering IOP.

Further studies of other types of collagens present in the TM are necessary to elucidate the role of lack of synthesis of ECM components in the outflow pathway and their effect on the IOP elevation mechanism.

## CONFLICT OF INTERESTS

The authors declare that they have no competing interests.

## AUTHOR CONTRIBUTION

**Saray Tabak:** Data curation (lead); Formal analysis (lead); Investigation (equal); Methodology (equal); Visualization (equal); Writing‐original draft (equal); Writing‐review & editing (equal). **Sofia Schreiber‐Avissar:** Conceptualization (lead); Funding acquisition (equal); Project administration (lead); Resources (lead); Supervision (equal); Validation (equal); Writing‐review & editing (lead). **Elie Beit‐Yannai:** Conceptualization (lead); Funding acquisition (lead); Investigation (equal); Methodology (equal); Project administration (lead); Resources (equal); Supervision (equal); Validation (equal); Visualization (equal); Writing‐review & editing (lead).

## ETHICAL APPROVAL

Not applicable.

## CONSENT FOR PUBLICATION

Not applicable.

## Supporting information

Appendix

## Data Availability

The data sets during and/or analysed during the current study available from the corresponding author on reasonable request.
